# Development of an Enhanced System Review for Public Agency Multi-Sectoral Integrated Data Systems

**DOI:** 10.23889/ijpds.v9i5.2876

**Published:** 2024-09-18

**Authors:** Baron Rodriguez

**Affiliations:** 1 WestEd/DISC

While traditional security assessments (such as mandated external security reviews which include a penetration test tied to a security framework) are a critical component to securing your data infrastructure, the complexities and governance structures around integrated data systems that involve multiple public sector agencies require a more comprehensive framework to address comprehensive risk, regulatory, and governance considerations that are critical to the confidentiality of the data within these systems.

In January of 2024, the Data Integration Support Center (DISC) created and piloted an enhanced system review that includes components that are often overlooked or oversimplified when working with today's technologies such as cloud-based secure enclaves. By following up on a mandated security review, an enhanced system review can help a maturing Integrated Data System (IDS) address both security and privacy risks as it moves forward. An enhanced system review looks primarily, though not necessarily exclusively, at the following aspects of an IDS, principally in the interest of ensuring privacy:

- IDS alignment with its legal framework and other structuring documents (e.g., policies, procedures, interagency agreements)
- Training to support privacy and security
- Use of technology to prevent or mitigate human error

The results of the review are provided directly to the IDS leadership and generally include a list of recommendations in the areas of improved legal agreements, leveraging privacy enhancing technologies to mitigate or reduce errors, procedural/policy gap identification and/or architectural recommendations to improve the security.

